# A pH-dependent shift of redox cofactor specificity in a benzyl alcohol dehydrogenase of aromatoleum aromaticum EbN1

**DOI:** 10.1007/s00253-024-13225-z

**Published:** 2024-07-08

**Authors:** Yvonne Gemmecker, Agnieszka Winiarska, Dominik Hege, Jörg Kahnt, Andreas Seubert, Maciej Szaleniec, Johann Heider

**Affiliations:** 1 Laboratory for Microbial Biochemistry, Philipps University of Marburg, 35043 Marburg, Germany; 2 Jerzy Haber Institute of Catalysis and Surface Chemistry, Polish Academy of Sciences, Niezapominajek 8, 30-239 Krakow, Poland; 3 Mass Spectrometry and Proteomics, Max Planck Institute for Terrestrial Microbiology, Marburg, Germany; 4 Faculty of Chemistry, Analytical Chemistry, Philipps-University Marburg, Marburg, Germany; 5 Center for Synthetic Microbiology, Marburg, Germany

**Keywords:** Benzyl alcohol dehydrogenase, Anaerobic benzyl alcohol degradation, Zn-dependent, Enzyme kinetics, Aromatic alcohols

## Abstract

**Abstract:**

We characterise a reversible bacterial zinc-containing benzyl alcohol dehydrogenase (BaDH) accepting either NAD^+^ or NADP^+^ as a redox cofactor. Remarkably, its redox cofactor specificity is pH-dependent with the phosphorylated cofactors favored at lower and the dephospho-forms at higher pH. BaDH also shows different steady-state kinetic behavior with the two cofactor forms. From a structural model, the pH-dependent shift may affect the charge of a histidine in the 2′-phosphate-binding pocket of the redox cofactor binding site. The enzyme is phylogenetically affiliated to a new subbranch of the Zn-containing alcohol dehydrogenases, which share this conserved residue. BaDH appears to have some specificity for its substrate, but also turns over many substituted benzyl alcohol and benzaldehyde variants, as well as compounds containing a conjugated C=C double bond with the aldehyde carbonyl group. However, compounds with an sp^3^-hybridised C next to the alcohol/aldehyde group are not or only weakly turned over. The enzyme appears to contain a Zn in its catalytic site and a mixture of Zn and Fe in its structural metal-binding site. Moreover, we demonstrate the use of BaDH in an enzyme cascade reaction with an acid-reducing tungsten enzyme to reduce benzoate to benzyl alcohol.

**Key points:**

*•Zn-containing BaDH has activity with either NAD* ^*+*^ *or NADP*^*+*^ *at different pH optima.*

*•BaDH converts a broad range of substrates.*

*•BaDH is used in a cascade reaction for the reduction of benzoate to benzyl alcohol.*

**Supplementary Information:**

The online version contains supplementary material available at 10.1007/s00253-024-13225-z.

## Introduction

*Aromatoleum aromaticum* strain EbN1 belongs to the order *Rhodocyclales* in the class Betaproteobacteria and grows on many different substrates under either aerobic or denitrifying conditions (Rabus and Widdel 1995; Rabus et al. 2019). It accepts various aromatic or aliphatic substrates, e.g. several amino acids, organic acids, aldehydes, ketones or alcohols. Most of the respective degradation pathways involved have been identified in recent years, and many of the participating enzymes have been biochemically characterised (Rabus and Heider 1998; Rabus et al. 2005; Wöhlbrand et al. 2007; Lahme et al. 2014; Schühle et al. 2016; Arndt et al. 2019; Rabus et al. 2019; Schmitt et al. 2019). The genome sequence is available (Rabus et al. 2005), and the genes coding for most of the respective enzymes have been identified along with their regulation in the presence or absence of the respective substrates (Rabus and Widdel 1996; Wöhlbrand et al. 2007; Rabus et al. 2014; Vagts et al. 2021). However, the functions of few annotated gene products remain elusive. In contrast to the related species *Thauera aromatica* (Biegert et al. 1996), *A. aromaticum* EbN1 grows on benzyl alcohol as sole substrate (Rabus and Widdel 1995). The degradation pathway is initiated by oxidation to benzaldehyde via a benzyl alcohol dehydrogenase (Schmitt et al. 2019), which was proposed to be encoded by the gene *ebA3166* (CAI07904) on the basis of sequence similarity (Rabus et al. 2005; Wöhlbrand et al. 2007). However, proteomic studies did not show enhanced expression of this gene in cells grown on benzyl alcohol, but an apparent constitutive production of the gene product at low levels (Wöhlbrand et al. 2007). Therefore, we set out to identify its function by cloning the gene and characterizing the encoded protein biochemically.

Alcohol dehydrogenases (ADHs) (E.C. 1.1.1.x), which belong to the oxidoreductase superfamily, catalyze the interconversion between alcohols and aldehydes or ketones with high stereoselectivity under mild conditions by consuming the cofactor nicotinamide adenine dinucleotide or its phosphate (NAD^+^/NADP^+^) (Jörnvall et al. 2010; Nealon et al. 2015). This group of enzymes consists of several unrelated enzyme families, which vary in their activities, active site structures and amino acid sequences. ADHs are present in all areas of life, best understood are eukaryotic examples like the horse liver alcohol dehydrogenase (8ADH) (Colonna-Cesari et al. 1986; Plapp et al. 2017), or yeast alcohol dehydrogenase (5ENV) (Plapp et al. 2016), but there are examples from extremophiles like archaea (5YVM) (Ma and Tse 2015; Grötzinger et al. 2018; Akal et al. 2019) and from several bacteria (e.g. 1F8F, 5TNX, 1RJW, 6TQD, 4EEZ) (Reid and Fewson 1994; Landete et al. 2008; Shrivastava et al. 2011; Uthoff and Steinbüchel 2012). Most of the known ADHs are currently affiliated to three major families: family I consists of the medium- or long-chain zinc-containing ADHs, the most studied group in vertebrates; family II, the short-chain ADHs without metal cofactor; and family III, the iron-dependent or iron-activated enzymes (Conway et al. 1987; Ingram et al. 1987; Reid and Fewson 1994; Hernández-Tobías et al. 2011). The alleged benzyl alcohol dehydrogenase of *A. aromaticum* is affiliated to the long-chain ADHs of family I, which contain a catalytic Zn^2+^ ion in the active center. This catalytic Zn^2+^ is usually ligated in a tetraedic geometry involving two cysteines, a histidine and a glutamate as highly conserved ligands. If all four residues bind the Zn^2+^, the enzymes are in an open, catalytically incompetent conformation, which is assumed to facilitate the exchange of redox cofactors. To attain catalytic competence, the enzymes shift to a closed conformation with inversion of the Zn^2+^-liganding geometry by dissolving the Zn^2+^-glutamate bond while retaining the bonds to the other ligands. In this state, the fourth binding site of Zn^2+^ is occupied by a water or the oxygen atom of a substrate molecule (Guntupalli et al. 2021). In addition, most family I ADHs carry a second structural metal ion (mostly another Zn^2+^) ligated by four conserved cysteines (Jörnvall et al. 2015; Plapp et al. 2017). The reaction is initiated by binding the hydroxyl group of the alcohol (or the oxo group of the aldehyde) to the catalytic Zn^2+^ ion, either by exchange with the water ligand in the closed state or by binding to Zn^2+^in the open state with simultaneous inversion and replacing the glutamate ligand (Guntupalli et al. 2021). The Zn^2+^ then promotes deprotonation of the hydroxy group due to its Lewis acidity, and this triggers the transfer of a hydride from the C-atom carrying the hydroxylate group to the nearby located NAD(P) cofactor, as revealed by structural studies, pH dependencies and chemical principles (Karlsson et al. 2003; Eklund and Ramaswamy 2008; Pennacchio et al. 2013; Jörnvall et al. 2015; Guntupalli et al. 2021). ADHs are involved in a multitude of pathways in vivo/in nature, e.g. alcohol or hydrocarbon degradation, alcohol production, detoxification and catalysing partial steps in many complex pathways (Nnyepi et al. 2007; Hernández-Tobías et al. 2011; Keller et al. 2013; Ma and Tse 2015; Dong et al. 2018).

Recent biotechnological approaches employed ADHs for catalyzing asymmetric reactions (An et al. 2019), biocatalytic cascade reactions with reduced environmental impact and shortened synthesis routes compared to the classical counterparts (Dudzik et al. 2015; Keller et al. 2017; Dong et al. 2018; Borowiecki et al. 2020). Furthermore, ADHs are often employed as biocatalysts for the dynamic kinetic resolution of racemic substrates and for the preparation of enantiomerically pure chemicals (Musa and Phillips 2011; Borowiecki et al. 2020). Here, we report the properties of a promiscuous zinc-dependent benzyl alcohol dehydrogenase (BaDH) from *Aromatoleum aromaticum* EbN1 accepting either NAD- or NADP-based redox cofactors, which is affiliated to a novel branch within ADH family I. Furthermore, we demonstrate its potential use in a coupled enzyme reaction for the direct reduction of acids to alcohols with a whole cell system providing tungsten-containing aldehyde oxidoreductase as auxiliary enzyme (Arndt et al. 2019; Winiarska et al. 2022; Winiarska et al. 2023).

## Materials and methods

### Recombinant gene expression, preparation of cell-free extracts and enzyme purification

The *bdh* gene of *A. aromaticum* strain EbN1 (*ebA3166*; CAI07904, (Rabus et al. 2005)) was amplified via PCR from chromosomal DNA using the primers 5′-AAGCTCTTCAATGAAGATTCAAGCCGCAGTAAC-3′ (forward) and 5′-AAGCTCTTCACCCGGCGAGCCTAGGACCGGC-3′ (reverse). The PCR product was cloned into a broad-host range variant (Salii et al. 2021) of the vector pASG-IBA103 (IBA Lifesciences, Göttingen, Germany) following the manufacturer’s instructions. The resulting plasmid codes for a fusion protein of BaDH with a C-terminal Twin-Strep-tag (Schmidt et al. 2013). BaDH was subsequently produced in *E. coli* BL21(DE3) which was grown in LB medium supplemented with 2 % (v/v) ethanol at 37 °C and induced at room temperature with added anhydrotetracycline as reported previously (Schühle et al. 2016). Cells were harvested by centrifugation (15 min at 5000 rpm and 6 °C), resulting in 5 to 6 g wet cell mass/L of medium, which was stored frozen at − 80 °C. For preparing the enzyme, cells were resuspended in three volumes of 100 mM Tris/HCl, 150 mM KCl pH 7.5, 10 % glycerol containing 0.1 mg/ml DNase I and 0.1 mg/ml lysozyme. Cell-free extracts were prepared by sonification at 4 °C for 15 min, followed by ultracentrifugation (100,000 × g, 60 min). BaDH activity was exclusively observed in the soluble fraction. Cell-free extracts with overproduced BaDH were applied on a Strep-tactin® affinity column (IBA Lifesciences, Göttingen, Germany), and further purification of the protein was performed as reported before (Schühle et al. 2016). The identity of the produced recombinant protein was checked from the masses of tryptic fragments, using a 4800 Proteomics Analyzer (MDS Sciex,Concord, ON, Canada). MS data were evaluated against an in-house database using Mascot embedded into GPS explorer software (MDS Sciex, Concord, ON, Canada) (Arndt et al. 2019). Recombinant production of the tungsten enzyme aldehyde oxidoreductase (AOR) from *A. aromaticum* was done using the closely related species *A. evansii* as host as described (Winiarska et al. 2023).

### Protein chemical methods

The purified protein was analysed by SDS-PAGE (12 % polyacrylamide; Laemmli 1970) and native polyacrylamide gel electrophoresis as described (Gallagher 2018). The molecular mass of native BaDH was determined by applying the proteins to a calibrated gel filtration column (Superdex 200 PG, calibration kit HMW, GE Healthcare), by a Ferguson plot analysis with native gels (6–10 % polyacrylamide; NativeMark, Thermo Fischer) and by crosslinking analysis after treatment with glutaraldehyde or dimethylsuberimidate, as described previously (Schühle et al. 2016). Metal contents of protein fractions and controls were analyzed by inductively coupled plasma mass spectrometry (ICP-MS) as described previously (Arndt et al. 2019); protein concentrations were determined as described in Bradford (1976). Analysis of metal content of purified protein and respective buffer solutions was performed as described previously (Arndt et al. 2019).

### Enzymatic assays and HPLC analysis for product confirmation

BaDH activity was assayed in 100 mM Tris/HCl buffer (initially at pH 8.0 for alcohol oxidation and at pH 6.5 for aldehyde reduction) in a photometric assay by directly following the formation of NADH at 340 nm (ε = 6.22 mM^−1^ cm^−1^) at 30 °C. The assay contained purified recombinant BaDH (5-10 μg/ml), 1 mM NAD^+^ or 0,75 mM NADH respectively. The reactions were started by adding the substrate of interest (benzyl alcohol 0–3.5 mM; benzaldehyde 0–2.5 mM). The assays for establishing the pH optima of BaDH were performed with 50 mM citrate buffer between pH 5.0 and 5.5, 50 mM K-phosphate buffer between pH 6.0 and 8.0 and 50 mM Tris–Cl buffer between pH 8.5 and 9.0. The results were evaluated using the Enzyme Kinetic package of the Origin Pro 2022. Three types of models best fitted the data:

1. ‘Bell shape’ model:
  $$V=\frac{V_{\textrm{lim}}^{\textrm{max}}}{\left(1+{10}^{pK_a- pH}+{10}^{pH-{pK}_b}\right)}$$
2. ‘Bell shape with plateau’ model
  $$V=\frac{V_{\textrm{lim}}^{\textrm{max}}\left(\alpha +{10}^{pK_b- pH}\right)}{\left(\left(1+{10}^{pK_a+{pK}_b-2\ast pH}\right)+{10}^{pK_b- pH}+{10}^{pH-{pK}_c}\right)}$$
3. ‘Plateau shaped’ model:
  $$V=\frac{V_{\textrm{plateau}}^{\textrm{max}}+{V}_{\textrm{lim}}^{\textrm{max}}\ast {10}^{pH-{pK}_b}}{\left(1+{10}^{pK_a- pH}+{10}^{pH-{pK}_b}\right)}$$

The best respective model was selected based on adjusted values of *R*^2^ and χ^2^.

Later on, assays were performed at the respective optimal pH conditions for every combination of substrate and redox cofactor: benzyl alcohol oxidation with NAD^+^ at pH 8.0, but with NADP^+^ at pH 6.0, and benzaldehyde reduction with NADH at pH 7.0, but with NADPH at pH 5.5. Assays with concentrations of > 0.5 mM NAD(P)H were measured at 375 nm, using an experimentally determined *ε*_375_ = 1.19 mM^−1^ cm^−1^, and those with concentrations of > 2 mM were measured in 0.5 or 0.2 cm glass cuvettes. Enzyme assays for steady state kinetic analysis were evaluated by using GraphPad Prism.

To confirm aldehyde reduction by BaDH, a reaction mixture in 100 mM K-phosphate buffer pH 6.5, containing 0.75 mM NADH and 15 μg/ml purified enzyme was incubated at 30 °C and started by adding benzaldehyde (0.8 mM). For product confirmation of alcohol oxidation, a reaction mixture in 100 mM Tris–Cl buffer pH 7.8, containing 0.75 mM NAD^+^ and 15 μg/ml purified enzyme was prepared, and the reaction was started by adding benzyl alcohol (1.3 mM). The reactions were followed spectrophotometrically at 365 nm, and samples were taken at *t* = 0 and *t* = 1 min of reaction time. The reactions were stopped by mixing the collected samples with acetonitrile in an 1:1 (v/v) ratio. The concentrations of both reactants, benzyl alcohol and benzaldehyde, were then measured by HPLC on a 1260 UHPLC-DAD instrument (Agilent) using a ZORBAX 300 SB-C18 column (RRHD, 2.1 × 50 mm, 1.8-micron, Agilent, USA), which was eluted at 30 °C at a flow rate of 0.2 ml/min in isocratic mode with a 0.1% formic acid in a H_2_O/ACN 65/35 mobile phase (2 μl injection volume). Benzyl alcohol was detected at 210 nm for and eluted at an RT of 1.9 min, while benzaldehyde was detected at 250 nm and eluted at an RT of 4.3 min. The quantitation of both compounds was conducted with external standard calibration (see Fig. S1-3 in the Supplementary Information). The samples collected from cascade reaction experiments were analyzed at 30 °C at a flow rate of 0.4 ml/min in isocratic mode with a 0.1% formic acid in a H_2_O/ACN 75/25 mobile phase (2 μl injection volume).

### Coupled assay with AOR

A cascade reaction system involving purified BaDH together with the isolated tungsten enzyme aldehyde oxidoreductase (AOR) from *A. aromaticum* was already reported to afford the direct reduction of benzoic acid to benzyl alcohol with H_2_ as sole reductant, as previously described in Winiarska et al. (2022). In this work, we used a whole-cell system of recombinant *A. evansii* cells producing AOR together with purified BaDH added to the cell suspension together with substrate and redox cofactor. The reactor tests were conducted in a volume of 3.0 ml of 50 mM citrate buffer pH 5.5 with adding cell suspensions of *A. evansii* cells containing recombinant AOR of either 0.54 or 0.6 g (wet mass) ml^−1^, as well as 0.28 mg/ml of purified BaDH and 1 mM NADH. The reactions were started with the addition of substrate (sodium benzoate) to an end concentration of 100 mM. Reactions were run under an anaerobic atmosphere of N_2_/H_2_ (97.5:2.5), and the solutions were preincubated in this H_2_-containing atmosphere for at least 30 min prior to the reaction. As controls, the reaction was conducted with 0.54 g of the recombinant cells aerobically in the absence of H_2_ or withouth cells containing AOR. The reaction progress was followed for formation of benzyl alcohol by HPLC over a period of 4 h using HPLC-DAD analysis. At each time point, two independent samples were collected, and the reaction was stopped by mixing the samples with acetonitile in a 2:1 (v/v) ratio. Each sample was analyzed in triplicate.

### Phylogenetic analysis

The amino acid sequences of BaDH and those of various other members of the Zn-containing ADH family were aligned using Clustal Omega (www.ebi.ac.uk/Tools/msa/clustalo and avermitilis.ls.kitasato-u.ac.jp/clustalo) with bootstrap values calculated in 1000 replications. A neighbour-joining tree was constructed based on the alignment, using the Program iTOL (itol.embl.de/).

### AlphaFold2 model

The model of BaDH was obtained from AlphaFold Protein Structure Database while the Zn ion and the NADH cofactor were copied from BaDH of *A. calcoaceticus* (PDB code 1F8F) after superimposing both structures in PyMol 2.5.2 (Schrodinger, LLC). The phosphate group at the C2′ atom of NADH was added in Discovery Studio Visualizer (Biovia, v.18), and its geometry was optimised with a clean geometry tool.

## Results

### Production and purification of recombinant benzyl alcohol dehydrogenase

**Fig. 1:**
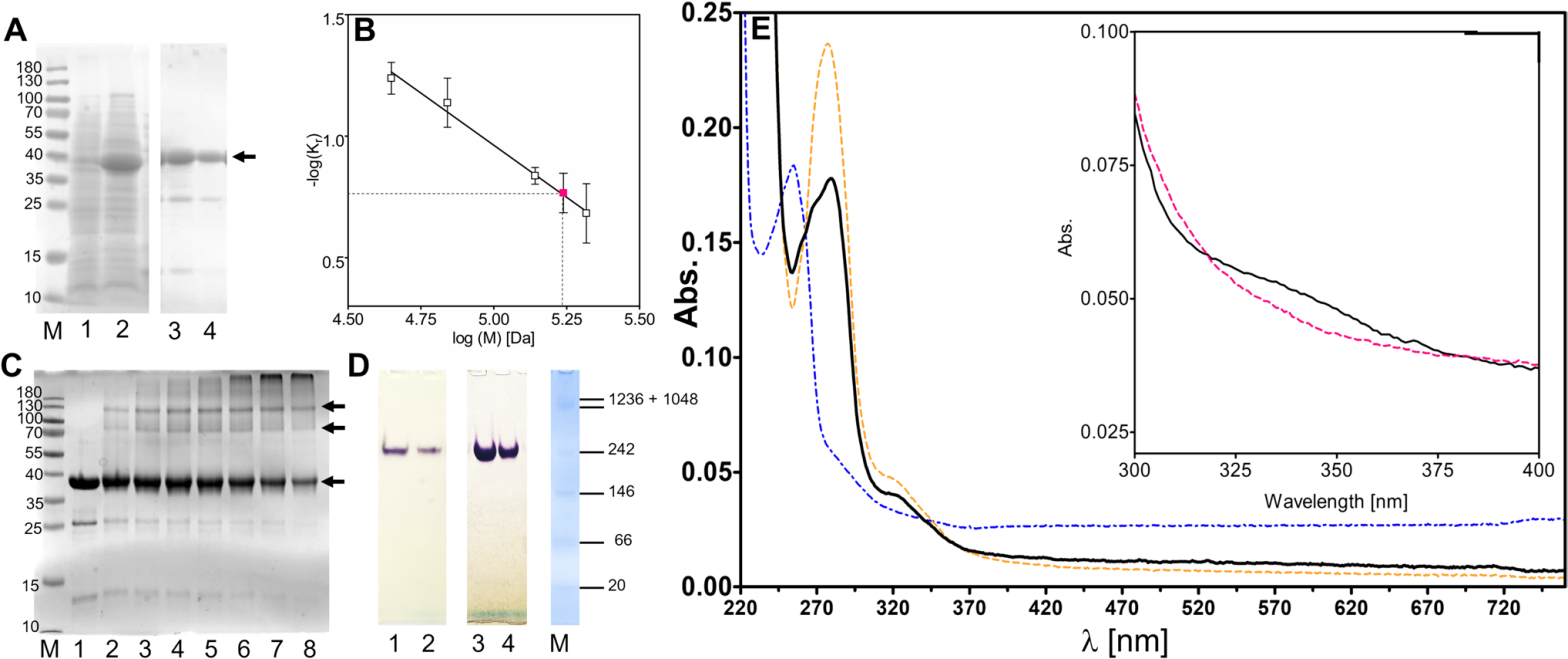
Molecular and spectroscopic properties. **A** SDS-PAGE analysis of protein fractions. Lanes: M marker proteins; 1, extract of uninduced cells; 2, extract of induced cells; 3 and 4; fractions after affinity chromatography. The arrow indicates the purified protein at 41 kDa. **B** Ferguson plot analysis: ovalbumin of bovine serum albumin oligomers are indicated as white, BaDH as red squares. **C** Crosslinking analysis. Lanes: M marker proteins, 1, BaDH before crosslinking, 2–8, after 1, 3, 5, 7, 10, 12 and 15 min of crosslinking. Arrows indicate the positions of the predicted monomer, dimer and tetramer. **D** Activity staining result. Lanes: 1, 2, extracts of *A. aromaticum* EbN1 grown on benzyl alcohol or benzoate, respectively; 3, 4, fractions of purified recombinant BaDH; M native PAGE markers. **E** UV-Vis spectrum of recombinant BaDH. Spectra are as follows: solid black line, enzyme as purified; dotted blue line, benzyl alcohol standard, broken orange line, difference spectrum of benzyl alcohol-treated BaDH and benzyl alcohol standard. The insert shows the enlarged region between 300 and 400 nm with protein before (black line) and after treatment with H_2_O_2_ (magenta dashed line)

The gene *ebA3166* of *A. aromaticum* EbN1 (accession number CAI07904; (Rabus et al. 2005)) was amplified via PCR and cloned into the expression plasmid pASG103 via the StarGate cloning system (IBA Lifesciences, Göttingen, Germany). The plasmid contained the gene with an added motif coding for an added C-terminal Twin-Strep-tag to facilitate purification of the protein. Expression was induced in recombinant *E. coli* cultures by adding 200 ng/ml AHT. The protein was initially formed in the form of inclusion bodies, but adding 2% ethanol to the medium resulted in producing it as a soluble protein. Ethanol induces the production of chaperones in *E. coli*, which have been reported to prevent inclusion of body formation in some cases (Thomas and Baneyx 1997). After lysing the harvested cells and ultracentrifugation, the cell extract indeed contained benzyl alcohol dehydrogenase (BaDH) activity and an overproduced soluble protein of the expected size (41 kDa). The enzyme was purified in one step via Strep-affinity chromatography and tested for NAD^+^-dependent benzyl alcohol oxidation, yielding a specific activity of 71 U (mg protein)^−1^ with a total enzyme activity of 1050 U and total protein content of 14.8 mg from 10 g (wet mass) of recombinant *E. coli* cells. The enzyme appeared essentially pure with only minor contaminations after SDS-PAGE analysis (Fig. 1A).

### Molecular properties of benzyl alcohol dehydrogenase

The native molecular mass of BaDH was determined as 164 kDa by gel filtration and Ferguson plot analysis (Fig. 1B), while the theoretical molecular mass of the subunit was confirmed as 41.4 kDa by SDS-PAGE (Fig. 1A). This suggests that the enzyme forms a homotetrameric complex under native conditions, as was also confirmed by crosslinking experiments, which produced bands corresponding to the sizes of the dimers and tetramers (Fig. 1C). Elemental analysis revealed the presence of 1.34 atoms of zinc and 0.37 atoms of iron per subunit of BaDH, indicating that the enzyme carries Zn in the catalytic and a mixture of Zn and Fe in the structural metal-binding site. The amino acids involved in binding these metals in other members of the family are completely conserved in BaDH: Cys40, His60 and Cys166 act as permanent ligands of the catalytic Zn^2+^ with Glu61 as additional ligand of a potential open form and Thr42 as conserved second-shell ligand, and a conserved CX_2_CX_2_CX_7_C motif with Cys89, 92, 95 and 103 binds the structural metal ion, probably either Zn^2+^ or Fe^2+^ in case of BaDH (Guntupalli et al. 2021). The only other elements found in significant amount were phosphorus (0.22 per subunit) and nickel (0.01 per subunit; Table S1).

### Correlation to BaDH activity in benzyl alcohol-degrading *A. aromaticum* EbN1

To test whether the recombinantly produced BaDH is identical with the enzyme used for benzyl alcohol degradation in *A. aromaticum* EbN1, we compared the behavior of BaDH in cell extracts of *A. aromaticum* with that of recombinant BaDH by separating the proteins by native polyacrylamide electrophoresis and staining for BaDH activity (Fig. 1D). The extracts of *A. aromaticum* EbN1 cells grown on benzyl alcohol or benzoate showed a common single band after activity staining for benzyl alcohol–oxidising activity, confirming a previous report based on proteomic analysis that the *ebA3166* gene product is not induced in benzyl alcohol–grown cells, but constitutively expressed at low level (Wöhlbrand et al. 2007). The purified recombinant protein could not be analysed in the same gels because of the large differences in activity, but showed a very similar migration behavior in activity-stained native gels, strongly suggesting that it represents the same protein. The migration positions of native and recombinant proteins showed a slight deviation, as must be expected because of the attached Strep-tag fusion (Fig. 1D).

### Spectroscopic features

Interestingly, the amino acid sequence of BaDH does not contain any tryptophan and only its 4 tyrosines and 17 phenylalanines contribute as chromophores at 280 nm, resulting in predicted extinction coefficients of 5.96 mM^−1^ cm^−1^ without and 16.96 mM^−1^ cm^−1^ with the two tryptophan residues present in the double Strep-tag added to the recombinant BaDH. Surprisingly the UV-Vis spectrum of the recombinant BaDH displays two unusual characteristics (Fig. 1E). Next to the expected peak at 280 nm, two additional shoulders appear at approximately 260 nm and 330 nm (Fig. 1E). The small absorption feature at 330 nm is reminiscent to spectra reported for the reduced form of rubredoxins (Yoon et al. 1999). Therefore, it may be related to the detected iron, which is most probably bound in about half of the subunits as Fe^2+^ by the four cysteine ligands in the structural metal-binding site which resembles the rubredoxin metal cluster. After treating the protein with excess H_2_O_2_, this absorbance disappeared without significant change of any other peaks, suggesting oxidative removal of the Fe^2+^ from the protein (Fig. 1E). The additional absorbance at 260 nm may be correlated with the measured substoichiometric P content, which might indicate the presence of a bound nucleotide or a similar compound. After acidifying the protein by adding HCl and removing the denaturated protein by centrifugation, we indeed detected small amounts of an extracted compound in the supernatant which showed a mass peak of 541 Da in MS analysis. Unfortunately, we did not obtain the compound in sufficient amounts for further characterisation. Another surprising observation was that the spectrum changed after adding of benzyl alcohol, exhibiting increased absorption at 280 nm and loss of the additional absorption at 260 nm (Fig. 1E). After calculating an experimental extinction coefficient *ε*_280_ from the two spectra, the originally prepared form of BaDH exhibited a value of 13 mM^−1^cm^−1^ for *ε*, while the benzyl alcohol-treated form showed a value of 17 mM^−1^ cm^−1^, placing the latter very close to the theoretically expected value.

### Reaction parameters of BaDH

**Fig. 2:**
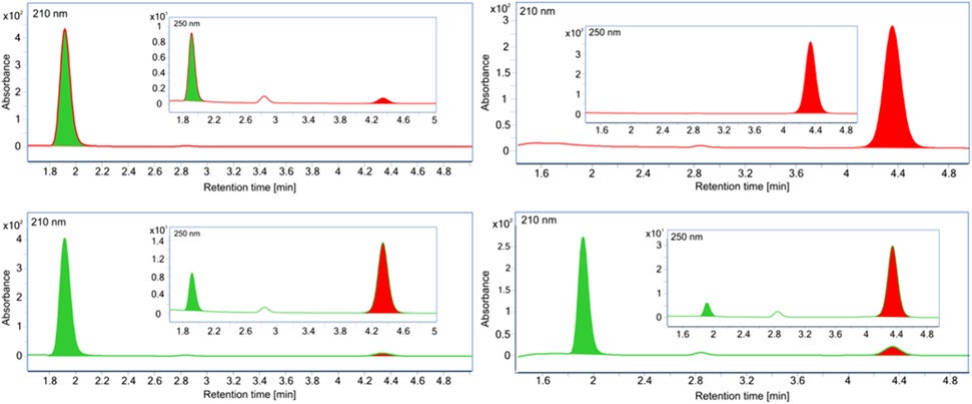
HPLC analysis of the reaction products. Retention times: benzyl alcohol, 1.9 min (labeled in green); benzaldehyde, 4.3 min (labeled in red); top chromatograms show starting conditions, the bottom ones the products after 1-min turnover. Left: benzyl alcohol oxidation assay; right: benzaldehyde reduction assay. Benzyl alcohol was usually detected by absorption at 210 nm, benzaldehyde at 250 nm; the traces at both wavelengths are shown with absorption at 250 nm in the insets

We determined the pH and temperature optima for the catalyzed alcohol oxidation (forward) and aldehyde reduction (reverse) reactivities and assayed both NAD and NADP as redox cofactors. Surprisingly, BaDH showed good activities with either cofactor, both in the forward and reverse direction. Formation of the desired products was confirmed by HPLC analysis (Fig. 2). Reduction of benzaldehyde at pH 7.5 with excess NADH resulted in converting 91.3% of the aldehyde to benzyl alcohol, whereas less than 10% of benzyl alcohol was oxidised to benzaldehyde in the forward reactions with excess NAD^+^, leading to similar equilibrium benzaldehyde/benzyl alcohol ratios of 0.095 and 0.105, respectively.

**Fig. 3:**
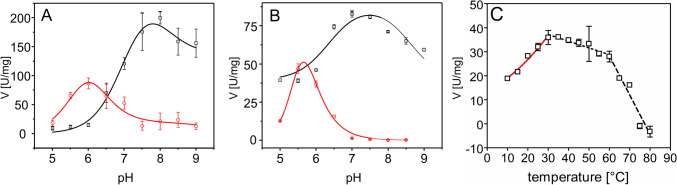
pH and temperature dependence of BaDH. **A** pH dependence of benzyl alcohol oxidation with NADP^+^ (red) or NAD^+^ (black). **B** pH dependence of benzaldehyde reduction with NADPH (red) or NADH (black). **C** Temperature dependence of benzyl alcohol oxidation with NAD^+^. The red curve indicates the part fitted to the Arrhenius equation; the two regions exhibiting linear decrease are indicated by black broken lines

Surprisingly, analyzing the pH dependence of BaDH with either redox cofactor revealed two completely different pH optima: NADP^+^ or NADPH was optimally turned over at slightly acidic conditions with the fastest recorded rates at pH 6.0 for benzyl alcohol oxidation and at pH 5.5 for benzaldehyde reduction. Curve fitting revealed best fits with a bell-shaped model for the former and a bell-shaped model with plateau for the later data set with calculated pH optima at pH 6.2 and 5.5, respectively (Fig. 3; see Tables S3 and S4 for fitting parameters and Fig. S4 and S5 for all data). In contrast, the highest reaction rates with NAD^+^ or NADH were under slightly alkaline conditions at pH 8.0 for benzyl alcohol oxidation and pH 7.0 for benzaldehyde reduction. Curve fitting showed best fits with a plateau model for the former and a bell-shaped model for the later data set with almost identical calculated optima at pH 7.5 (Fig. 3; see Tables S5 and S6 for fitting parameters and Fig. S6 and S7 for all data).

The temperature dependence of the reaction was measured for benzyl alcohol oxidation with NAD^+^ at pH 8.0. The plot revealed an exponential increase of enzyme activity with temperature up to 30 °C, then a slow linear decrease between 30 and 60 °C, and a faster decline at higher temperatures until the activity is lost at 75 °C (Fig. 3C). Non-linear fitting of the initial values to the Arrhenius equation reveals an activation energy value of 25.8 ± 1.5 kJ/mol (see Table S7 for all parameters).

### Substrate spectrum of BaDH

To investigate the substrate spectrum of BaDH, we performed the enzyme activity assay described above with different substrates. Depending on the availability of the various substrates, we either tested for alcohol oxidation with NAD^+^ or for aldehyde reduction with NADH. Some substrates of interest, which are not soluble in the required concentrations under aqueous conditions, were prepared as stock solutions in tert-butanol before adding them to the assay. Therefore, we verified that BaDH neither reacts with tert-butanol nor is inhibited by it. The data show that BaDH is quite specific for benzyl alcohol resp. benzaldehyde. BaDH is also active with their substituted derivatives carrying one or more hydroxy-, methoxy-, amino-, alkyl-, nitro- or chloro-substituents on the aromatic ring, albeit at lower, but still quite significant rates (Table 1; see also Table S2 for structures). Additional substrates accepted by BaDH are benzylic alcohols and aldehydes with heteroaromatic rings, such as furfural, or unsaturated alcohols and aldehydes carrying a conjugated double bond between C2 and C3, e.g. crotonaldehyde or 3-phenyl-2-propen-1-ol (cinnamic alcohol). Significantly lower or no activity has been observed with substrates carrying a sp^3^-hybridised methylene group at C2, such as 2-phenylethanol or 3-phenylpropanol, or with secondary alcohols or ketones like 1-phenylethanol or α-vinylbenzyl alcohol (Table 1; see also Table S2 for structures). Theses data suggest that the substrates need to provide simultaneously a primary alcohol or aldehyde group and either an aromatic ring or a double bond in conjugation with the carbonyl group of the aldehyde (substrate or product) to be turned over at high rates by BaDH.

**Table 1:** Substrate spectrum of BaDH in alcohol oxidation with NAD^+^ or aldehyde reduction with NADH. Assays were done at 30 °C in 100 mM Tris/HCl buffer (pH 8.0) containing purified BaDH and 1 mM NAD^+^ or 0.75 mM NADH for alcohol oxidation or aldehyde reduction, respectively. The reactions were started by adding 1 mM of the respective substrates and followed photometrically at 340 nm. Relative activities refer to the benzyl alcohol-oxidising (rA^BAlc^) or benzaldehyde-reducing activity (rA^BAld^) of the same batch of enzyme. SA specific activity [U/mg]

Alcohol oxidation with NAD^+^			Aldehyde reduction with NADH		
Substrate	SA [U/mg]	rA^BAlc^ [%]	Substrate	SA [U/mg]	rA^BAld^ [%]
Benzyl alcohol	289	100	Benzaldehyde	22.2	100
α-vinylbenzylalcohol	0.25	0.86	4-hydroxybenzaldehyde	1.29	5.83
3-benzyloxy-2-methylpropan-1-ol	0.01	0	4-isopropylbenzaldehyde	9.60	43.3
2-phenylethanol	0.01	0	4-nitrobenzaldehyde	16.5	74.6
Rac-1-phenylethanol	0.01	0	4-chlorobenzaldehyde	11.2	54.1
3-phenyl-2-propen-1-ol	17.2	59.3	2-aminobenzaldehyde	0.37	1.68
3-phenyl-1-propanol	0.05	0.17	4-hydroxy-3-methoxybenzaldehyde	4.56	20.6
4-hydroxybenzylalcohol	24.1	83.2	(2*E*)-but-2-enal	1.57	7.08
4-methoxybenzylalcohol	10.5	36.29	furan-2-carbaldehyde	9.87	44.5
2-(4-hydroxyphenyl)ethanol	0	0	4-fluorobenzaldehyde*	137*	106*
Phenol	0.04	0	pyridine-3-carbaldehyde*	86.9*	67.3*
2-methylpropan-2-ol	0.04	0	(2E)-3-phenylprop-2-enal*	46.7*	38.5*
1-ethylcyclohexanol	0	0	Octanal*	4.79*	3.71*
3-methyl-3-oxetane methanol	0	0	Butanal*	2.26*	1.75*
4-chloro-1-naphthol	0	0	Phenylacetaldehyde*	0	0*
ethanol	0	0	Acetaldehyde*	0	0
methanol	0	0			

*Assays performed with a different enzyme batches with considerably higher specific activity

### Reaction kinetics

**Fig. 4:**
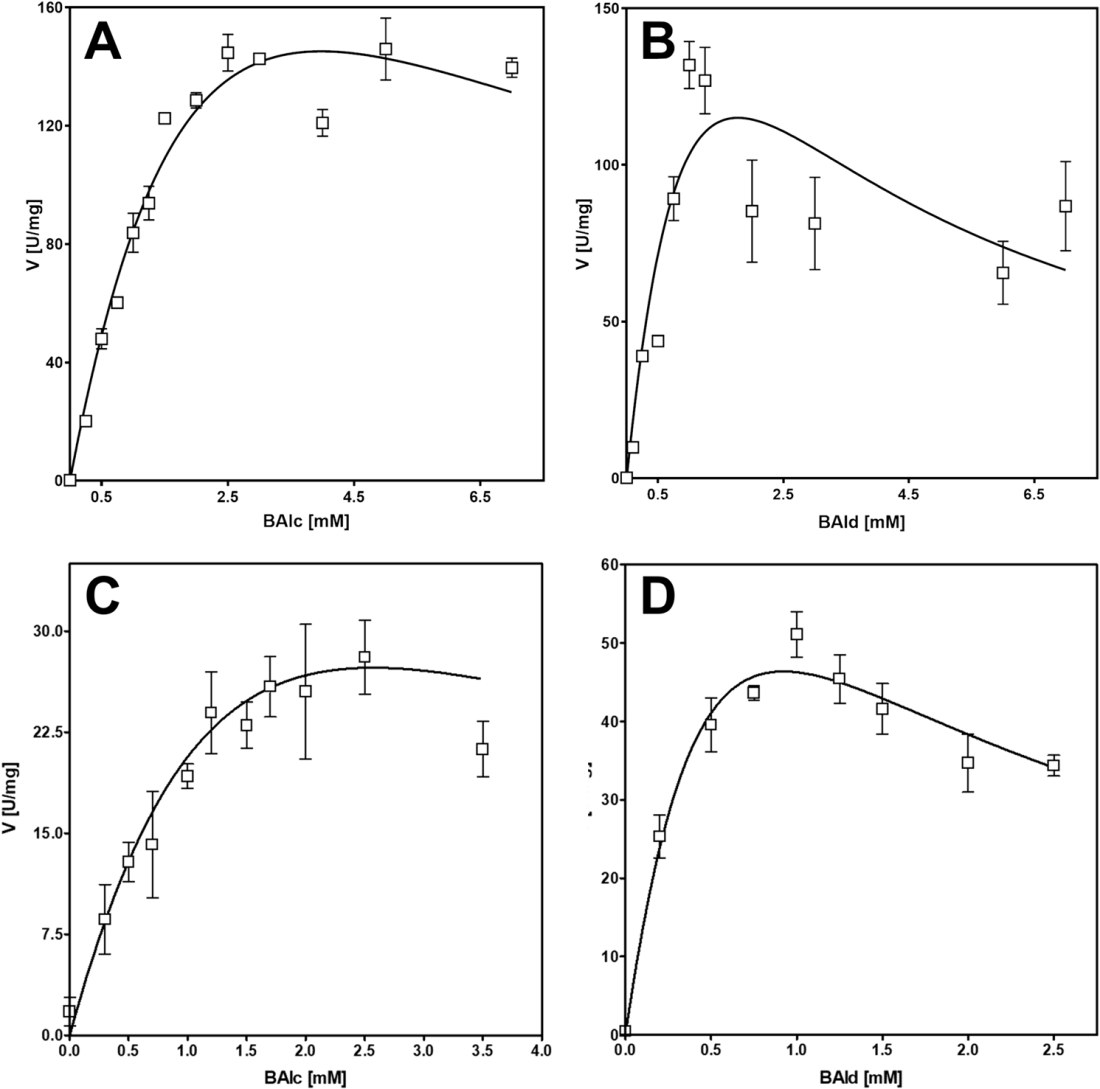
Steady-state kinetics analysis of benzyl alcohol (BAlc) and benzaldehyde (BAld) conversion with BaDH. **A** Oxidation of benzyl alcohol with NAD^+^ at pH 8.0. **B** Reduction of benzaldehyde with NADH at pH 7.0. **C** Oxidation of benzyl alcohol with NADP^+^ at pH 6.5. **D** Reduction of benzaldehyde with NADPH at pH 5.5

As BaDH showed benzyl alcohol oxidation activity with either NAD^+^ or NADP^+^ and benzaldehyde-reducing activity with either NADH or NADPH at different pH optima, we determined the respective apparent steady-state parameters for the substrates as well as for the redox cofactors under these conditions. For benzyl alcohol and benzaldehyde, the enzyme followed Michaelis-Menten kinetics with substrate inhibition, which depended in its extent on the redox cofactor used. The assays with NAD^+^ and NADH showed almost linear activity increase up to 1 to 2 mM of the substrates, but turned to saturation and eventually substrate inhibition beyond these concentrations (Fig. 4A, B). The kinetics of both benzyl alcohol and benzaldehyde conversion were therefore fitted against the Michaelis-Menten equation with integrated substrate inhibition. For benzyl alcohol oxidation, curve fitting yielded calculated *K*_*m*_ and *K*_is_ values of 3.6 and 4.4 mM, and a theoretical *V*_max_ value of 410 U mg^−1^, although the highest actually measured rates were in the range of 140 U mg^−1^ (Fig. 4A, B). To obtain a meaningful data set for the curve fitting data of benzaldehyde reduction, *K*_*is*_ was constrained to be larger or equal than *K*_*m*_, yielding values of *K*_*m*_ = *K*_*is*_ = 1.78 mM and a *V*_max_ = 345 U mg^−1^, which was again much higher than the highest measured rate of about 130 U mg^−1^ (Fig. 4A, B). In case of the assays with benzyl alcohol/NADP^+^ and benzaldehyde/NADPH, we recorded significantly lower activities for both substrates, but they also showed strong substrate inhibition, which required constraining *K*_*is*_ and *K*_*m*_ during curve fitting (Fig. 4C, D). Because of the applied constraints, we obtained identical values for *K*_*m*_ and *K*_*is*_ together with much higher theoretical *V*_max_ values than the rates actually measured: the parameters for benzyl alcohol oxidation were *K*_*m*_ = *K*_*is*_ = 2.6 mM with *V*_max_ = 82 U mg^−1^, those for benzaldehyde reduction were *K*_*m*_ = *K*_*is*_ = 0.9 mM and *V*_max_ = 139 U mg^−1^, while the highest measured rates were at 25 and 50 U mg^−1^, respectively. The apparent overestimation of all theoretical *V*_max_ values is caused by the very strong observed substrate inhibition with *K*_*is*_ values very close to the respective *K*_*m*_ values (or even significantly lower without using constraints). The quality of the curve fits was assessed by inspecting the respective *R*^2^ values, which were still reasonable even after constraining the parameters. However, because of the strong discrepancy between theoretical *V*_max_ values and the actually measured rates, we refrain from using the respective *k*_cat_ parameters for describing the enzyme reactions (Table 2).

**Table 2:** Summary of the apparent kinetic parameters of benzyl alcohol oxidation and benzaldehyde reduction with both forms of nicotinamide nucleotides. Substrate kinetics: *BAlc ox* oxidation of benzyl alcohol, *BAld red* reduction of benzaldehyde, using the respective cofactor. Cofactor kinetics: *NAD(P) red* reduction of NAD(P)^+^, *NAD(P)H ox* oxidation of NAD(P); models: *MM* Michaelis-Menten, *MM S*_*i*_ Michaelis-Menten with substrate inhibition, *Hill S*_*i*_ Hill model with substrate inhibition

	*V*_max_ [U/mg]	*K*_*m*_ [mM]	*k*_cat_ [s^−1^]	*n*	*K*_*is*_ [mM]	Fit model	*R*^2^	*k*_cat_/*K*_*m*_ [mM^−1^ s^−1^]
BAlc ox NAD^+^ pH 8.0^a^	4.10 x10^2^	3.62	6.93 x10^2^	n.a.	4.37	MM S_i_	0.95	191
BAlc ox NADP^+^ pH 6.5^a^	81.8	2.57	56.7	n.a.	2.56	MM S_i_	0.71	22
BAld red NADH pH 7.0^a^	3.45 x10^2^	1.78	2.39 x10^2^	n.a.	1.78	MM S_i_	0.79	134
BAld red NADPH pH 5.5^a^	1.39 x10^2^	0.92	96.4	n.a.	0.92	MM S_i_	0.89	104
NAD^+^ red pH 8.0	84.2	1.67	58.4	n.a.	n.a.	MM	0.91	35
NADP^+^ red pH 6.5^a^	54.1	2.39	37.5	2.44	2.39	Hill S_i_	0.91	16
NADH ox pH 7.0^b^	3.20 x10^2^	3.69	2.22 x10^2^	n.a.	n.a.	MM	0.92	60
NADPH ox pH 5.5^a^	1.59 x10^2^	3.42	1.10 x10^2^	1.38	3.42	Hill S_i_	0.98	32

^a^Note that the calculated *V*_max_ and *k*_cat_ values are highly overestimated because of the closely similar values of the *K*_*m*_ and *K*_*is*_ parameters

^b^The activities in this experiment did not enter the saturation phase, because the required high NADH concentations were no longer distinguishable in the photometer. The data represent an extrapolation from the available data set we consider reliable, but probably underestimate the kinetic parameters

**Fig. 5:**
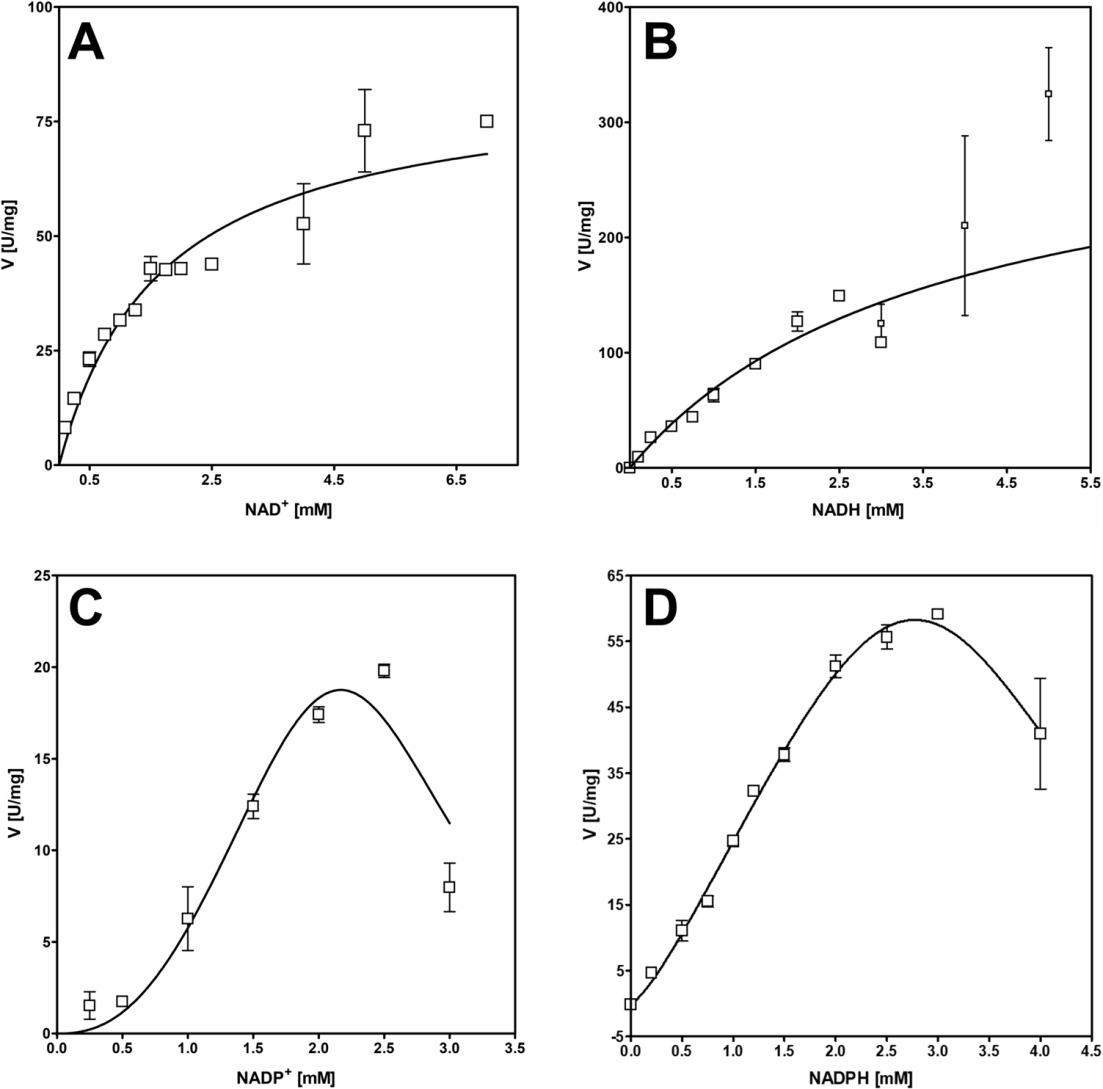
Steady-state kinetics analysis of the redox cofactors. **A** Oxidation of benzyl alcohol with NAD^+^ at pH 8.0; **B** reduction of benzaldehyde with NADH at pH 7.0 (small symbols represent measurements at absorption values of 0.7 to 2 at 375 nm with 0.2-cm cuvettes where the absorption decrease was hardly distinguishable from the background); **C** oxidation of benzyl alcohol with NADP^+^ at pH 6.5; **D** reduction of benzaldehyde with NADPH at pH 5.5

Analysis of the steady-state kinetics for the redox cofactors with fixed concentrations of the respective substrates revealed apparent cooperative kinetics with substrate inhibition for NADP^+^ and NADPH, but Michaelis-Menten kinetics for NAD^+^-dependent benzyl alcohol oxidation (Fig. 5A), which exhibited apparent *V*_max_ and *K*_*m*_ values of 84.2 U mg^−1^ and 1,67 mM NAD^+^. In contrast, no reliable kinetic constants for the NADH-dependence of benzaldehyde reduction were obtained, because the enzyme activity increased nearly linearly with the NADH concentrations, until the initial absorption values became so high that the reaction was no longer reliably represented, even by measuring at 375 nm and using thinner cuvettes. The best fits for NADP^+^-dependent benzyl alcohol oxidation and NADPH-dependent benzaldehyde reduction were obtained with a positively cooperative model with integrated substrate inhibition (Fig. 5C, D). As already observed for substrate inhibition of the substrates combined with the phosphorylated cofactors, the originally obtained *K*_*is*_ values were smaller than those for *K*_*m*_, requiring to constrain *K*_*is*_ > *K*_*m*_. Under these conditions, the fitting resulted in apparent values of *V*_max_ = 54.1 U mg^−1^, *K*_*m*_ = *K*_*is*_ = 2.39 mM of NADP^+^ for benzyl alcohol oxidation, and *V*_max_ = 159 U mg^−1^, *K*_*m*_ = *K*_*is*_ = 3.42 mM of NADPH for benzaldehyde reduction. Because of the similar values for *K*_*m*_ and *K*_*is*_, substrate inhibition sets in early, resulting in much higher theoretical *V*_max_ values than the maximum activities observed experimentally, which were in the range of 25 U mg^−1^ and 50 U mg^−1^, respectively (Fig. 5). As already noticed for the substrates, the obtained *K*_*m*_ values for the redox cofactors were unusually high, suggesting that BaDH is not saturated with either substrate or cofactor under *in vivo* conditions (see Table 2 for values).

### Application of BaDH in a coupled enzyme assay

**Fig. 6:**
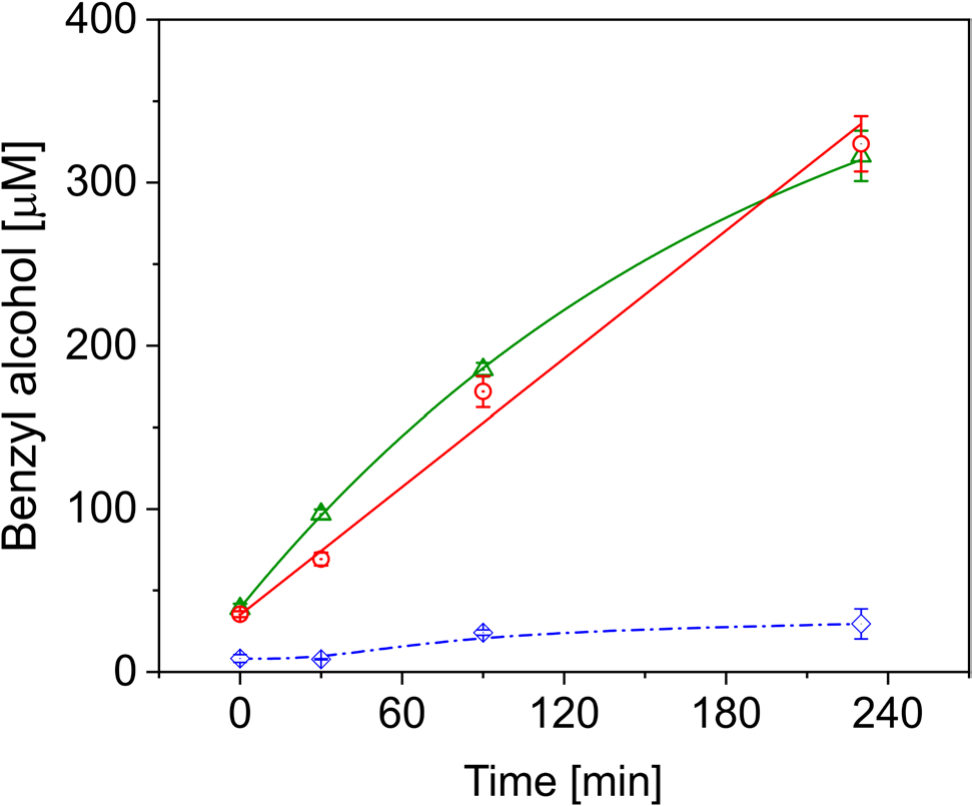
Reduction of benzoic acid to benzyl alcohol. A cascade reaction system comprised of whole cells of recombinant *A. evansii* with overproduced AOR, cell-free BaDH and NADH in the extracellular medium has been set up for H_2_-dependent reduction of benzoate to benzyl alcohol: red circles, 0.54 g ml^−1^ of cells; green triangles, 0.6 g ml^−1^ of cells; blue rhombi, aerobic control without H_2_ and with 0.54 g ml^−1^ of cells

In a previous study, we have already demonstrated that BaDH can be combined with the tungsten enzyme aldehyde oxidoreductase (AOR) from *A. aromaticum* for the H_2_-driven reduction of benzoic acid to benzyl alcohol via benzaldehyde (Winiarska et al. 2022). In this cell-free system, AOR directly utilises H_2_ for simultaneously reducing benzoic acid to benzaldehyde and NAD^+^ to NADH. These products are subsequently converted to benzyl alcohol by BaDH, recycling NADH back to NAD^+^. Because this system is limited by the considerable oxygen sensitivity of purified AOR, which is otherwise stable in cell extracts or whole cells (Arndt et al. 2019), we decided to test if the enzymatic coupling also works between BaDH and a whole-cell system of *A. evansii* cells with overproduced AOR from *A. aromaticum* (Winiarska et al. 2023). Since H_2_ should easily penetrate the cell membranes and the recombinant *A. evansii* cells were grown on benzoate, we expected that the substrates for the AOR reaction should have access to the enzyme in the cytoplasm. Therefore, we set up suspensions of whole cells with recombinantly produced AOR in a H_2_:N_2_ atmosphere (2.5%:97.5%) and added purified BaDH, benzoate and NADH, assuming that benzoate will first be taken up and reduced to benzaldehyde in the cytoplasm, then benzaldehyde will diffuse out of the cells and become reduced to benzyl alcohol by BaDH with NADH as electron donor. Indeed, we observed the production of more than 0.3 mM benzyl alcohol within a reaction time of 4 h under these conditions (Fig. 6). This equals 30% of the expected maximum, based on the available NADH added, and represents a doubled product yield compared to the best recorded cell-free system without any further optimisation. In the control experiments conducted under aerobic conditions, we obtained only 30 μM of benzyl alcohol. This indicates on one side that AOR still functions under these conditions, and on the other that the cells degrade benzoate aerobically and are still diverting some redox equivalents from that pathway towards AOR to simultaneously reduce some of the benzoate (Fig. 6). The second control reactor conducted without recombinant cells and containing only BaDH did not yield observable amounts of the product within the detection limit (data not shown). The outcome of these experiments proves that benzoate actually penetrates the cells of *A. evansii*, gets reduced to the aldehyde in the cytoplasm and diffuses back into the bulk solution where it is reduced to an alcohol. Therefore, further improvements can be expected with added NADH regeneration systems or by coexpressing the genes for both enzymes in *A. evansii*, which will provide intrinsic H_2_-dependent NADH recycling via AOR (Winiarska et al. 2022).

### Phylogeny

**Fig. 7:**
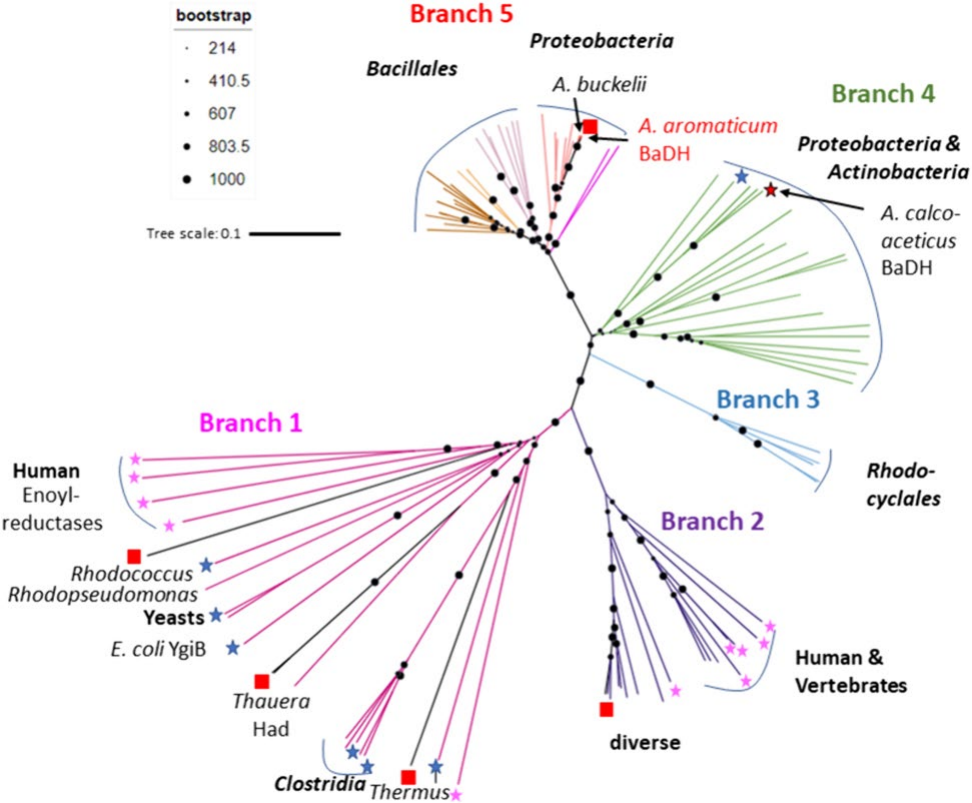
Phylogenetic tree of the Zn-containing alcohol dehydrogenase family. The phylogenetic affiliation of most clades is indicated. Entries with known structures are labeled with blue asterisks, those in magenta indicate human proteins, and proteins encoded in *A. aromaticum* are indicated by red squares. Note that the tree also covers family members that are not active as ADHs, such as the NADH-dependent enoyl-CoA or enoyl-ketone reductases of family I which have lost all residues involved in metal binding (Wu et al. 2008). Enzymes characterised as BaDH are located in branches 4 and 5 as indicated. Bootstrap values refer to an analysis with 1000 iterations

An initial BLAST search of *A. aromaticum* BaDH revealed that the protein is not closely related to other characterised enzymes of the zinc-dependent ADH family. Phylogenetic analysis with known representatives of the family and other related proteins from genome sequences revealed that the protein is part of a separate subbranch of the family together with several other predicted proteins from bacterial genomes (Fig. 7, branch 5). Although highly similar to each other, the members of branch 5 occur in bacteria of highly divergent phylogeny. In particular, the only other *Rhodocyclales* members coding for this enzyme are *A. aromaticum* pCyN1 and *A. buckelii*, which show almost 100% genome identity with *A. aromaticum* EbN1 (Rabus et al. 2019). The other enzymes of branch 5 are from the genomes of phylogenetically isolated species of Gamma- and Betaproteobacteria and mostly of Firmicutes affiliated to the *Bacillales*. Therefore, the gene has been most probably recently acquired by horizontal gene transfer. The closest relation to characterised enzymes is shared with a BaDH from *Acinetobacter calcoaceticus* (Gillooly et al. 1998; F1F8), which is affiliated to branch 4 of the family (Fig. 7). Branch 3 consists of uncharacterised enzymes from different *Aromatoleum* and *Thauera* species, whereas branches 1 and 2 contain most of the previously characterised members of the family, including other enzymes from *A. aromaticum* (Fig. 7).

## Discussion

We report here on BaDH from *A. aromaticum*, a new enzyme of the Zn-containing ADH family which catalyses oxidation of benzyl alcohol or reduction of benzaldehyde, but also accepts many other primary alcohols or aldehydes, but no secondary alcohols or ketones. In particular, the enzyme shows high activity with aromatic or heteroaromatic substrates, if the C-atom carrying the alcohol or aldehyde function is either directly attached to the aromatic ring or connected with it via conjugated double bonds (e.g. in cinnamic alcohol). In contrast, compounds containing saturated alkyl carbons between the aromatic ring and the terminal alcohol or aldehyde group are not or just barely turned over. Another type of substrates appears to be medium-chain aliphatic alcohols or aldehydes, which also seem to be turned over faster if they contain a C=C double bond in conjugation with the carbonyl group of the aldehyde. The catalysed reaction “benzyl alcohol + NAD(P)^+^ ⇆ benzaldehyde + NAD(P)H + H^+^” occurs with a calculated Gibbs-free enthalpy difference of ΔG°′ = 15.54 kJ mol^−1^ under standard conditions at pH 7.0, as calculated from the free enthalpies of formation (Thauer et al. 1977) and the redox potential of NADH/NAD^+^ of − 320 mV. This can be converted to benzaldehyde:benzyl alcohol ratios in the thermodynamic equilibrium of 1:23 at pH 7.0 or 1:8 at pH 8.0. These values fit remarkably well with those measured in the enzyme assays (performed at pH of 8.0) after reaching equilibrium, suggesting that the reaction has been running to completion under either oxidatve or reductive conditions. A remarkable feature of the enzyme is its ability to use either NAD^+^ or NADP^+^ (or the reduced counterparts) as redox cofactors. A more detailed analysis revealed that the use of the respective cofactor coincides with competely different pH profiles of the reactions. Using NADP^+^ or NADPH, the pH optima are in the acidic range (pH 6.0 and 5.5, respectively), while they are in the neutral to alkaline range for NAD^+^ or NADH (pH 8.0 and 7.0, respectively). Assays with the “wrong” cofactor at the pH optimum of the other revealed that the activities dropped to almost zero, except for benzaldehyde reduction with NADH at pH 5.5, which yielded almost equal activity as with NADPH. Taken together, these data indicate that redox cofactor selection by BaDH is strongly regulated by the cytoplasmic pH. Because the cellular pool of NADPH occurs almost competely in the reduced form, while NAD^+^ is mostly oxidised, this suggests an *in vivo* preference for benzaldehyde reduction at acidic and for benzyl alcohol oxidation at alkaline conditions. The preferred binding of NADP^+^ or NADPH under acidic conditions may also be connected with the need of binding the additional phosphate group at the C2′ atom. It is conceivable that the local charge content of the NAD(P) binding site exhibits a positively charged patch for NADP^+^ binding at lower pH values, but loses the charge by moving to higher pH values, allowing only NAD^+^ binding under these conditions.

**Fig. 8:**
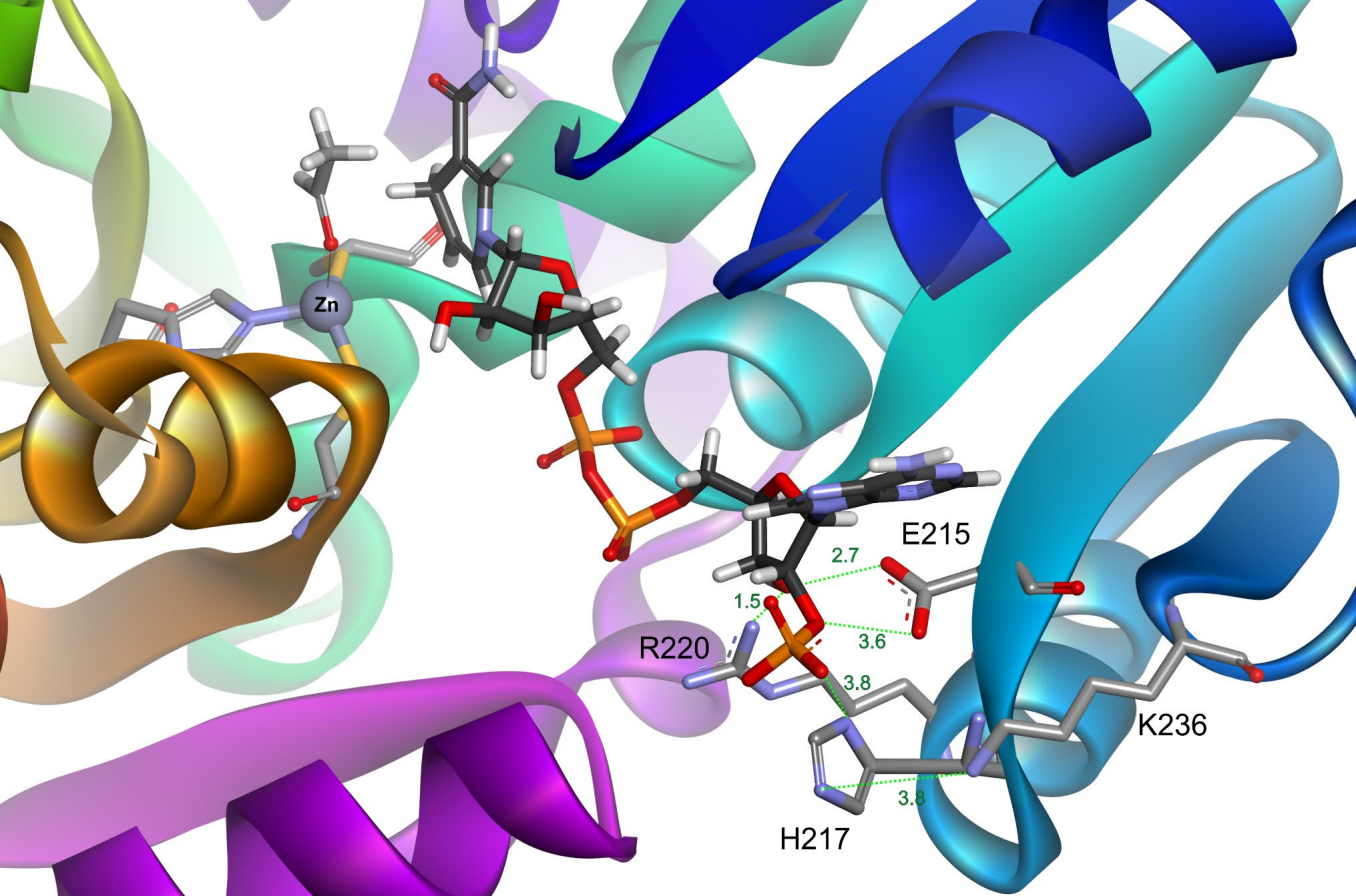
Detail from an AlphaFold model of BaDH. Bound NADPH and acetaldehyde are shown in the active site. The residues expected to be involved in binding the phosphoribosyl moiety and their distances [Å] to the targeted substituents of NADPH are shown without further optimisation of the model

Modelling the structure of BaDH using AlphaFold2 indeed predicts the binding of NAD^+^ or NADP^+^ in a very similar manner as known in other enzymes of the family (Fig. 8). In particular, the highly conserved residues Asp215 and Arg220, which are binding the 2′ and 3′ hydroxyl groups of NAD^+^ in ADHs in known structures (e.g. PDB 1F8F), are present in BaDH like in almost all other members of the family (Fig. 8). However, BaDH contains His217 as another auspicious residue in this domain, which would be ideally positioned to recognise the 2′-phosphate group of NADP^+^ (Fig. 8). This residue is strictly conserved in all enzymes of branch 5, but not in most other members of the family, including BaDH of *A. calcoaceticus*. Because the pK_a_ of histidine is close to the observed pH border for NAD^+^ or NADP^+^ specificity, we confidently expect that protonation or deprotonation of His217 is involved in redox cofactor switching. Another part of the pH-dependent switch may be Lys236 on the periphery of the structure, since it is ideally located to exchange protons with His217 and provides an easy proton relay to the solvent outside of the subunit (Fig. 8). Remarkably, Lys236 is not as conserved in the enzymes of branch 5 as His217, but still is present in most of the enzymes from the proteobacterial members and in about half of those from Firmicutes.

BaDH shows the usual homotetrameric composition of the Zn-containing ADH family, but differs in its metal content from the previously characterised enzymes. Instead of containing two Zn ions in a catalytic and a structural binding site per subunit, BaDH appears to have a Zn in the catalytic site and a mixture of Zn and Fe in the structural one, as suggested by metal analysis. In addition to maintaining the mechanistically important Zn rather than Fe in the catalytic site, the suggested presence of some Fe^2+^ in the structural metal binding site is supported by analysis of the UV-Vis spectra, because the small peak observed at 330 nm is remarkably similar to the spectrum of reduced rubredoxins (Ragsdale and Ljungdahl 1984; Yoon et al. 1999), which also contain a Fe^2+^ ligated by four cysteines. The observed loss of this absorption peak after treatment with H_2_O_2_ corroborates this prediction, suggesting that the oxidised Fe^3+^ form is no longer bound. The observed absorbance at 260 nm and the observed phosphorus content in purified BaDH suggest the presence of an additional nucleotide-like factor. Unfortunately, we were unable to identify this compound, since it was only obtained in quite limited amounts. The observed mass peak of 541 Da from the supernatant of the acid-denatured enzyme would fit to the mass of cyclic ADP-ribose (Lee et al. 1994), but this nucleotide has only been reported in eukaryotic cells (Banerjee et al. 2019), and no other common cytoplasmic nucleotide fits to this mass. An interesting feature is the observed restoration of the typical absorption peak at 280 nm by treatment with the substrate benzyl alcohol, suggesting that binding the unknown compound may convert BaDH to an inactive resting state which can be re-activated by substrate binding. This is in line with our observation that no BaDH activity could be measured spectrophotometrically in cell extracts of *A. aromaticum*, although the same extracts showed activity in activity staining (Fig. 1D), suggesting that the inhibitory compound was released during the native PAGE separation. Similarly, the overproduced enzyme in *E. coli* showed detectable activity in the extract, but the total enzyme activity increased about 10-fold after affinity purification, also suggesting the removal of an inhibitory compound during affinity purification.

Analysis of the temperature optimum of BaDH showed that the enzyme exhibits maximum activity at 30 °C, coinciding with the optimal growth temperature of *A. aromaticum* (Rabus and Widdel 1995). Its activity increases exponentially between 10 and 30 °C, following the Arrhenius equation which predicts a reasonable activation energy value of 25.8 kJ/mol. At higher temperatures, BaDH activity shows an unusually slow linear decrease pattern, still retaining 75% of its maximum activity at 60 °C, until it is rapidly inactivated and lost its activity completely at 70–80 °C. This behaviour suggests a complex mechanism of thermal deactivation or some rate compensation effects (e.g. change in the rate-determining step).

BaDH appears to change its steady-state kinetic properties depending on what redox cofactor is used. Using NAD^+^ or NADH, the enzyme shows Michaelis-Menten–like kinetics with substrate inhibition for both substrates and Michaelis-Menten kinetics for NAD^+^, albeit at unusually high *K*_*m*_ values. However, we we unable to determine reliable kinetics for NADH because the enzyme showed almost linear activity increase up to concentrations that were no longer measurable. Because of the unphysiologically high *K*_*m*_ values for the substrates and cosubstrates, it may be expected that BaDH shows an almost linear response to changes in substrate or redox cofactor concentrations in the physiologically available range. In contrast, the reactions coupled with NADP^+^ or NADPH appear to involve Michaelis-Menten–like kinetics for the substrates, but cooperative kinetics for the redox cofactors, both with added substrate inhibition, high apparent *K*_*m*_ and low *K*_*is*_ values. The kinetics for NADP^+^ or NADPH exhibit Hill coefficients of 2.44 and 1.38, respectively, suggesting that only binding of NADP^+^ or NADPH induces a cooperative conformation change of the quaternary structure to facilitate cofactor binding at the other subunits. This effect may help to somewhat counteract the rather high apparent *K*_*m*_ values (2.4 mM NADP^+^ and 3.4 mM NADPH), which are around 10-fold higher than their normal cytoplasmic concentrations. On the other side, the apparent lack of cooperativity of NAD^+^ or NADH binding may reflect their higher cellular abundance. The observed strong substrate inhibition by NADP^+^ and NADPH as well as the substrates may help to prevent disturbing the cytoplasmic NADPH/NADP^+^ ratio by limiting the attainable BaDH activity when high concentrations of substrate become available.

Phylogenetic analysis of BaDH based on its amino acid sequence indicates that the enzyme is part of a new branch of the Zn-containing ADH family and is not closely related to most previously reported ADHs. Because similar enzymes only occur in two highly related bacterial strains and several other unrelated Proteobacteria and Firmicutes, while being absent in many other more closely related species, we assume that its gene has only recently been acquired by horizontal gene transfer.

Finally, we show that BaDH may be applied as an auxiliary enzyme in synthetic pathways converting organic acids to alcohols, which may be useful as fine chemicals or biofuels. We have already shown previously that BaDH can be coupled with the tungsten enzyme AOR, which is directly reduced by H_2_ in these experiments, acting as H_2_-oxidising hydrogenase. Reduced AOR then simultaneously reduces benzoate to benzaldehyde and recycles NAD^+^ to NADH, which is required for BaDH to reduce benzaldehyde to benzyl alcohol (Winiarska et al. 2022). Because of the dual specificity of BaDH for NADH and NADPH, while AOR only accepts NAD^+^ (Arndt et al. 2019), we were able to perform the cascade reactions either with NADH recycling by AOR or with stoichiometric NADPH consumption by BaDH (Winiarska et al. 2022). In this study, we recorded the conversion of benzoate to benzyl alcohol in a coupled assay with BaDH and whole cells of *A. evansii* containing overproduced AOR and obtained higher yields of benzyl alcohol than in the cell-free system (Winiarska et al. 2022). Because of the broad substrate range of either AOR or BaDH, this conversion should also work with many other aromatic acids, and also with some medium-chain aliphatic acids (Arndt et al. 2019). Finally, we show that this conversion is not limited to anaerobic conditions and the presence of H_2_ as an electron donor for AOR, but works to some extent even aerobically without added H_2_. Therefore, the *A. evansii* cells must have shifted to degrade benzoate via the aerobic benzoyl-CoA pathway (Mohamed et al. 2001), deviating some electrons from there to benzoate reduction via AOR. Although the rate of benzyl alcohol production in this experiment was only about 10% of that in the anaerobic H_2_-dependent one, the observation shows that the coupled reaction is not oxygen-sensitive and may be optimised for alcohol production even without requiring H_2_ addition. Another convenient source of the electrons needed for acid reduction and NADH recycling by AOR would be an electrochemical cell. This principle has already been proven by showing electrochemical coupling of reactions catalysed by AOR (Kalimuthu et al. 2023) and by setting up an ATP-producing enzyme cascade reaction with AOR (Luo et al. 2023). Although the product concentrations produced in these assays are expected to stay relatively low even after further optimisation of the biological processes, some promising processes to concentrate alcohols from aqueous solutions with reasonably low energy demand are being developed, such as pervaporation (Golubev et al. 2018), membrane destillation (Alkhudhiri et al. 2012) or vapor-phase membrane filtration (Shalygin et al. 2023).

## Supplementary Information

ESM 1 (PDF 465 kb)

## Data Availability

The authors declare that the data supporting the findings of this study are available within the paper and its Supplementary Information files. Should any raw data files be needed in another format, they are available from the corresponding author upon reasonable request.
