# Supplementary material for: A pH-dependent shift of redox cofactor specificity in a benzyl alcohol dehydrogenase of aromatoleum aromaticum EbN1
Source: Appl Microbiol Biotechnol. 2024 Jul 8;108(1):410. doi: 10.1007/s00253-024-13225-z (PMC11231019; doi:10.1007/s00253-024-13225-z)
Supplement: Supplementary file 1 — (PDF 465 kb) [file 253_2024_13225_MOESM1_ESM.pdf]

Supplementary information

A pH-dependent shift of redox cofactor specificity in a benzyl alcohol dehydrogenase of *Aromatoleum aromaticum* EbN1

Yvonne Gemmecker[1], Agnieszka Winiarska[2], Dominik Hege[1], Jörg Kahnt[3], Andreas Seubert[4], Maciej Szaleniec[2*], Johann Heider[1,5]*

1 Laboratory for Microbial Biochemistry, Philipps University of Marburg, 35043 Marburg, Germany; e-mail@e-mail.com

2 Jerzy Haber Institute of Catalysis and Surface Chemistry, PAS, Niezapominajek 8, 30-239 Krakow, Poland; maciej.szaleniec@ikifp.edu.pl

3 Mass Spectrometry and Proteomics, Max Planck Institute for Terrestrial Microbiology

4 Faculty of Chemistry, Analytical Chemistry, Philipps-University Marburg, Marburg, Germany

5 LOEWE-Center for Synthetic Microbiology, Marburg, Germany

* Correspondence: heider@biologie.uni-marburg.de, Tel.: +49-6421-282-1527
* Correspondence: maciej.szalenie@ikifp.edu.pl, Tel.: +48-12-6395-218

Index

Methods 2
- Calibration curves of HPLC-DAD 2

Result 4
- Element analysis 4
- Substrates 5
- pH models – data and fit details 8
  - Oxidation of benzyl alcohol with NADP+ 8
  - Reduction of benzaldehyde with NADPH 9
  - Oxidation of benzyl alcohol with NAD+ 10
  - Reduction of benzaldehyde with NADH 11
- Temperature dependency of benzyl alcohol oxidation with NAD+ 12

Methods

Calibration curves of HPLC-DAD

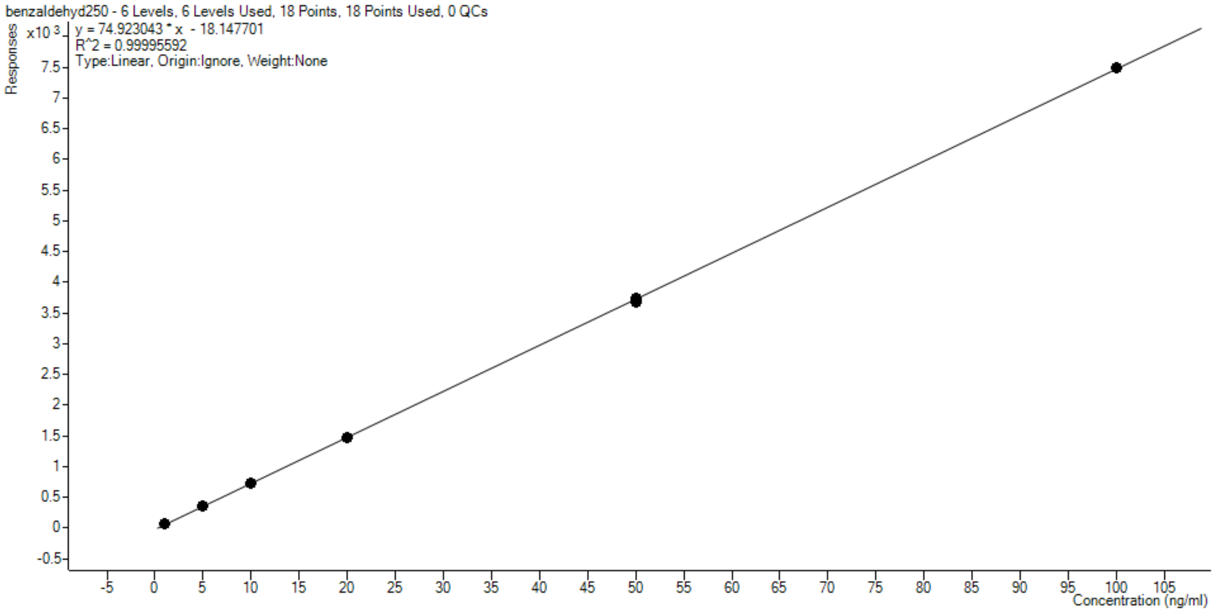

Fig. S1. Calibration curve for benzaldehyde (RT: 4.3 min); wavelength 250 nm.

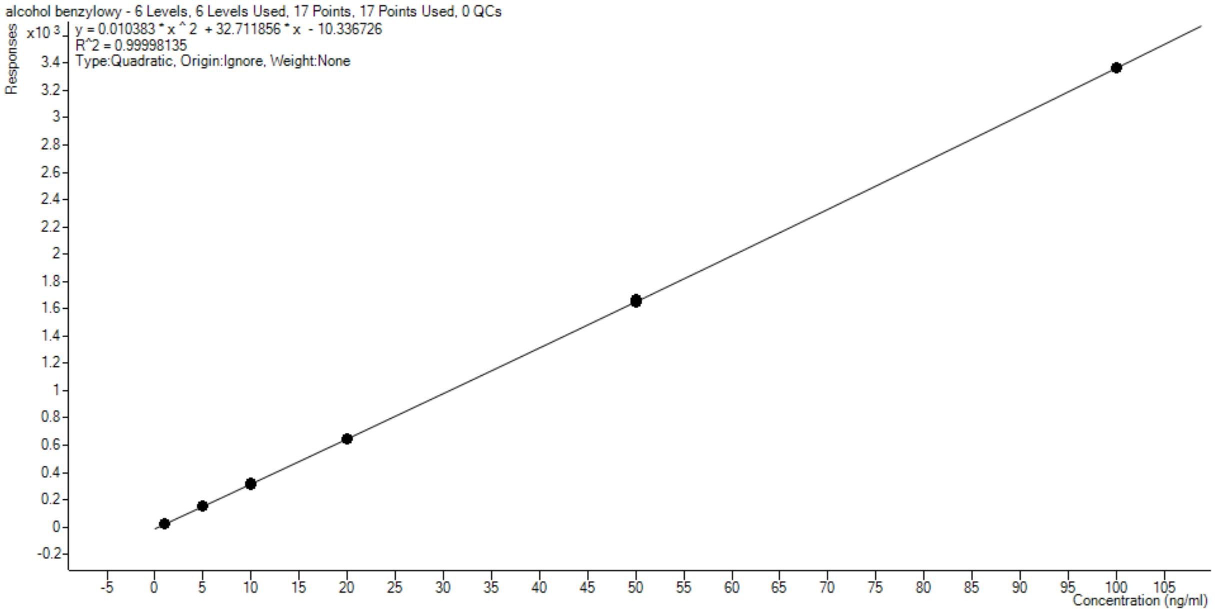

Fig. S2. Calibration curve for benzyl alcohol (RT: 1.9 min); wavelength 210 nm.

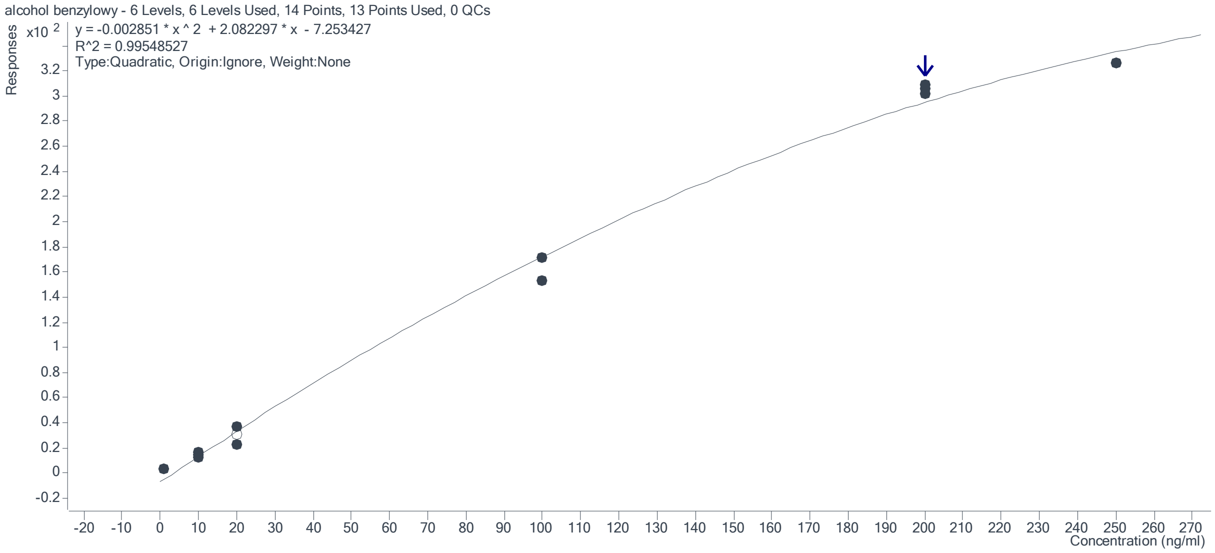

Fig. S3. Calibration curve for benzyl alcohol used in the analysis of cascade reaction (RT: 1.9 min); wavelength 210 nm.

Result

Element analysis

Table S1. Element analysis of BaDH. The element content is given as molar ratio per subunit after data correction for the empty buffer.

Element	Molar ratio
Mg	0.0015
P	0.221
Ca	< 0.02
Mn	< 0.001
Fe	0.370
Co	< 0.001
Ni	0.011
Cu	0.0018
Zn	1.34
Se	< 0.010
Mo	< 0.001
W	< 0.001

Substrates

Table S2.. Substrates used in the study: alcohols in NAD(P)$^+$ dependent oxidation and aldehydes in NAD(P)H dependent reduction

**Alcohols**		**Aldehydes**	
benzyl alcohol	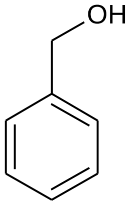	benzaldehyde	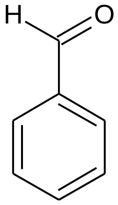
α-vinylbenzylalcohol	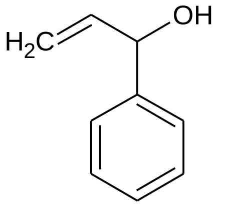	4-hydroxybenzaldehyde	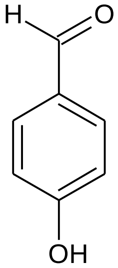
3-benzyloxy-2-methylpropan-1-ol	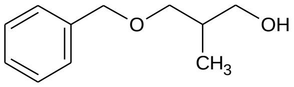	4-isopropylbenzaldehyde	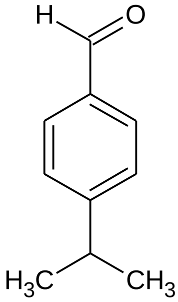
2-phenylethanol	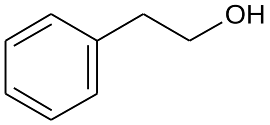	4-nitrobenzaldehyde	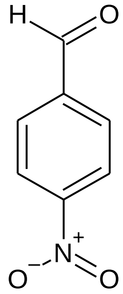
rac-1-phenylethanol	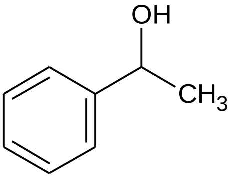	4-chlorobenzaldehyde	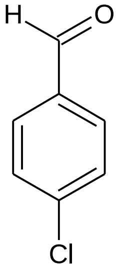
3-phenyl-2-propen-1-ol	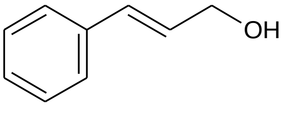	2-aminobenzaldehyde	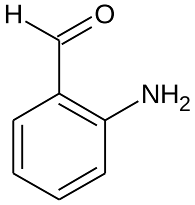

3-phenyl-1-propanol	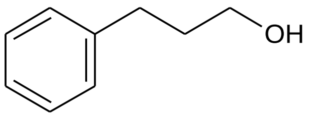	4-hydroxy-3-methoxybenzaldehyde	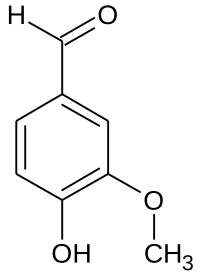
4-hydroxybenzylalcohol	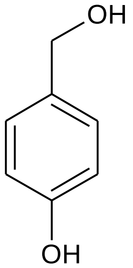	(2*e*)-but-2-enal	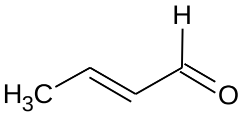
4-methoxybenzylalcohol	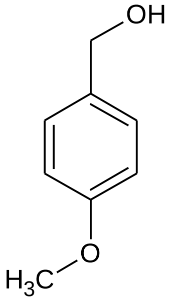	furan-2-carbaldehyde	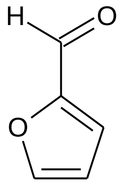
2-(4-hydroxyphenyl)ethanol	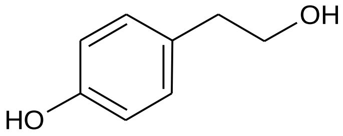	4-fluorobenzaldehyde	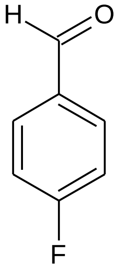
phenol	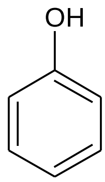	pyridine-3-carbaldehyde	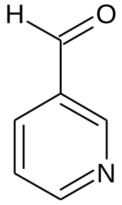
2-methylpropan-2-ol	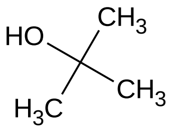	(2E)-3-phenylprop-2-enal	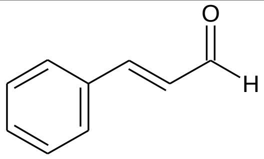
1-ethylcyclohexanol	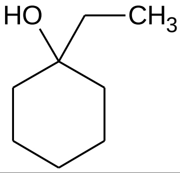	octanal	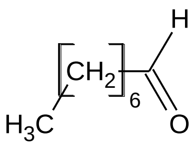

3-methyl-3-oxetane methanol	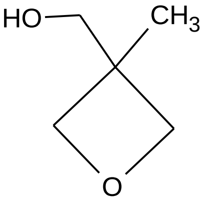	butanal	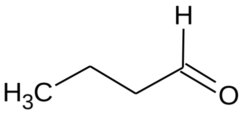
4-chloro-1-naphthol	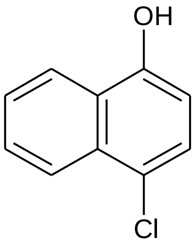	phenylacetaldehyde	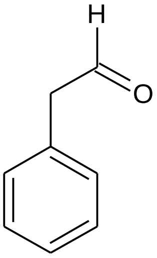
ethanol	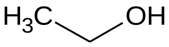	acetaldehyde	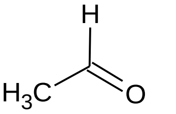
methanol	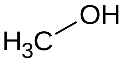		

pH models – data and fit details

Oxidation of benzyl alcohol with NADP+

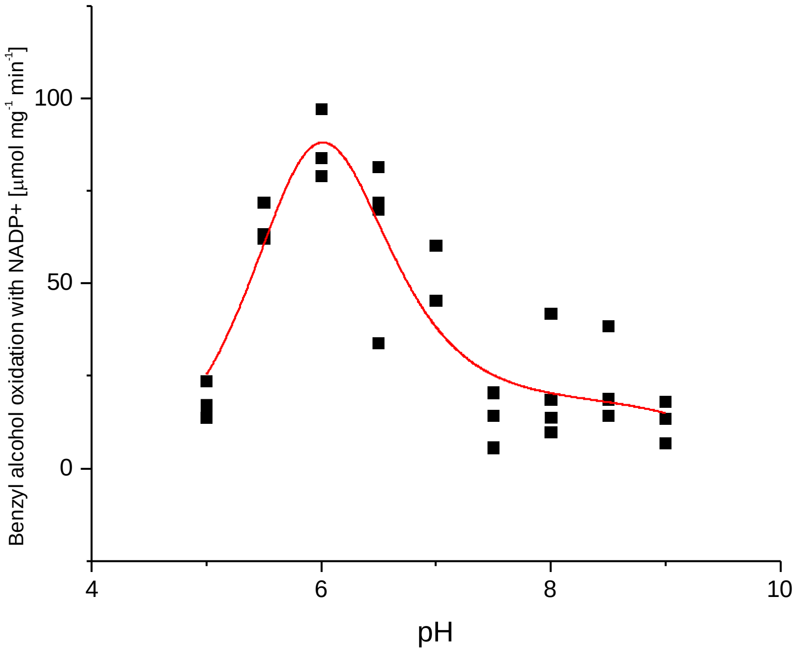

Fig. S4. pH dependence of benzyl alcohol oxidation with $NADP^+$ (black squares) with a 'bell-shaped' model fit (red line).

Table S3A. Statistical parameters non-linear regression of different models describing pH dependence of benzyl alcohol oxidation with $NADP^+$. Bold indicates the selected model.

	n	Degree of Freedom	Reduced χ^2	RSS	R^2	Adj. R^2
**Bell Shaped***	**28**	**25**	**265.8054**	**6645.135**	**0.69884**	**0.67475**
Bell Shaped With Plateau	28	23	176.3341	4055.685	0.8162	0.78423
Plateau Shaped	28	24	517.3855	12417.25	0.43725	0.36691

*The bell-shaped model was selected over the bell-shaped with plateau model due to more statistically reliable parameters (i.e. non-zero errors of estimates)

Table S3B. Fit parameters for pH dependence of benzyl alcohol oxidation with $NADP^+$. LCL lower confidence limit, UCL upper confidence limit

Model	Parameter	Value	Standard Error	95% LCL	95% UCL
	V^{max}_{lim}	113.03	23.65	64.32	161.73
	pK_a	5.44	0.23	4.97	5.92
Bell Shaped	pK_b	6.84	0.23	6.37	7.32

Reduction of benzaldehyde with NADPH

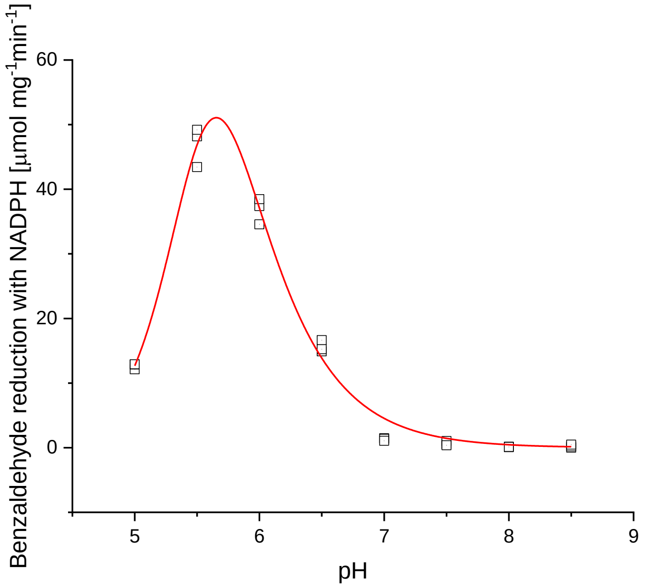

Fig. S5. pH dependence of benzaldehyde reduction with NADPH (squares) with a ‘plateau-shaped’ model fit (red line).

Table S4A. Statistical parameters non-linear regression of different models describing pH dependence of benzaldehyde reduction with NADPH. Bold indicates the selected model.

Model	n	Degree of Freedom	Reduced χ^2	RSS	R^2	Adj. R^2
Bell Shaped	24	21	121.76168	2556.99528	0.6359	0.60122
**Bell Shaped With Plateau**	**24**	**19**	**3.83221**	**72.81193**	**0.98963**	**0.98745**
Plateau Shaped	24	20	127.85222	2557.04438	0.63589	0.58128

Table S4B. Fit parameters for pH dependence of benzaldehyde reduction with NADPH. LCL lower confidence limit, UCL upper confidence limit.

Model	Parameter	Value	Standard Error	95% LCL	95% UCL
Bell Shaped With Plateau	V^{max}_{lim}	3.35	897.25	-1874.62	1881.32
	pK_a	5.50	19.85	-36.04	47.05
	pK_b	5.81	19.42	-34.83	46.45
	pK_c	5.13	10.25	-16.32	26.58
	α	102.13	29810.11	-62291.15	62495.41

Oxidation of benzyl alcohol with NAD$^+$

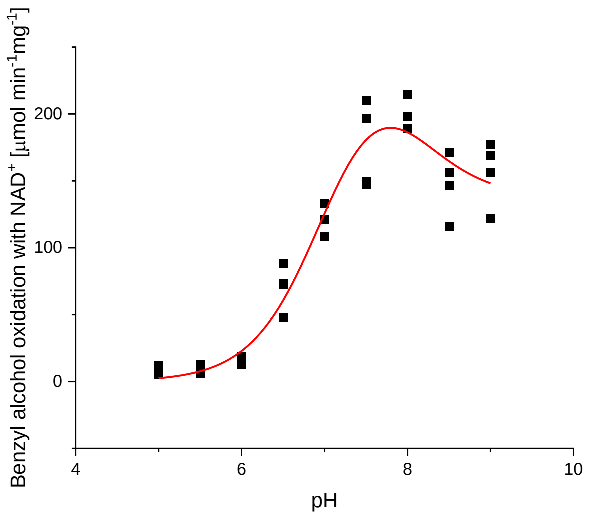

Fig. S6. pH dependence of benzyl alcohol oxidation with NAD$^+$ (black squares) with a 'plateau-shaped' model fit (red line).

Table S5A. Statistical parameters non-linear regression of different models describing pH dependence of benzyl alcohol oxidation with NAD$^+$. Bold indicates the selected model; RSS Residual Sum of Squares

Model	n	Degree of Freedom	Reduced χ^2	RSS	R^2	Adj. R^2
Bell Shaped	36	33	590.1	19473.68	0.89837	0.89221
Bell Shaped With Plateau	36	31	367.6	11394.24	0.94054	0.93286
**Plateau Shaped**	**36**	**32**	**356.1**	**11394.27**	**0.94054**	**0.93496**

Table S5B. Fit parameters for pH dependence of benzyl alcohol oxidation with NAD$^+$. LCL lower confidence limit, UCL upper confidence limit

Model	Parameter	Value	Standard Error	95% LCL	95% UCL
Plateau Shaped	$V^{max}_{plateau}$	250.52	37.85	173.42	327.61
	V^{max}_{lim}	137.39	16.28	104.23	170.55
	pK_a	7.00	0.14	6.71	7.29
	pK_b	8.03	0.44	7.13	8.93

Reduction of benzaldehyde with NADH

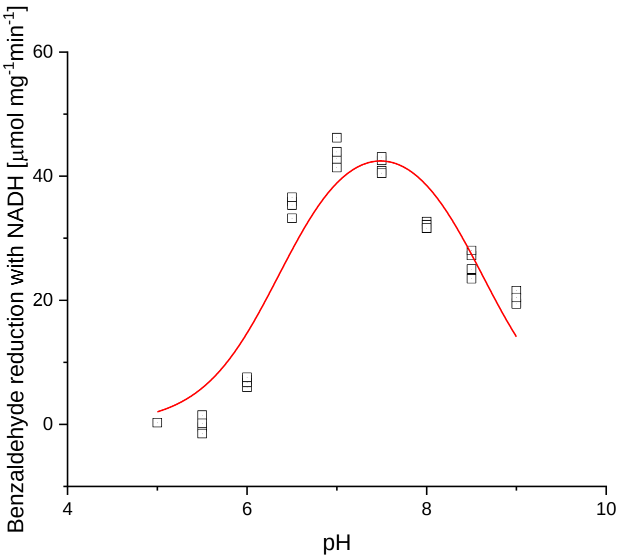

Fig. S7. pH dependence of benzaldehyde reduction with NADH (squares) with a ‘plateau-shaped’ model fit (red line).

Table S6A. Statistical parameters non-linear regression of different models describing pH dependence of benzaldehyde reduction with NADH. Bold indicates the selected model.

	n	Degree of Freedom	Reduced χ^2	RSS	R^2	Adj. R^2
**Bell Shaped**	**33**	**30**	**35.30**	**1058.92**	**0.86248**	**0.85331**
Bell Shaped With Plateau	33	28	12.21	341.86	0.9556	0.94926
Plateau Shaped	33	29	70.04	2031.22	0.73621	0.70893

*The values were corrected by the constant of -39.34 µmol mg^{-1} min^{-1}, i.e. minimal activity observed at pH 5.5 to enable convergence of the model which does not assume two plateaus. The second best ‘bell-shaped’ model was selected due to very high errors of estimates for $V^{max}{}_{lim}$ in the bell-shaped with plateau model.

Table S6B. Fit parameters for pH dependence of benzaldehyde reduction with NADH. LCL lower confidence limit, UCL upper confidence limit

Model	Parameter	Value	Standard Error	95% LCL	95% UCL
Bell Shaped	$V^{max}{}_{lim}$	88.19*	3.43	41.84	55.87
	pK_a	6.36	0.11	6.14	6.59
	pK_b	8.61	0.11	8.38	8.84

Corrected back by the constant of 39.34 µmol mg^{-1} min^{-1}

Temperature dependency of benzyl alcohol oxidation with NAD^+

Table S7. Statistical parameters non-linear regression of Arrhenius model describing temperature dependence of benzyl alcohol oxidation with NAD^+.

Model	n	Degree of Freedom	A [$U\ mg^{-1}$]	E^a [$J\ mol^{-1}$]	R^2
Arrhenius	19	17	4.13 $\times 10^5$	2.35 $\times 10^4$	0.88

Equation: Arrhenius $k = A \cdot e^{\frac{-E^a}{R \cdot T}}$
